# Genome-Wide Investigation of Genes Regulated by ERα in Breast Cancer Cells

**DOI:** 10.3390/molecules23102543

**Published:** 2018-10-05

**Authors:** Shuning Wang, Xiaoju Li, Wangqian Zhang, Yuan Gao, Kuo Zhang, Qiang Hao, Weina Li, Zhaowei Wang, Meng Li, Wei Zhang, Yingqi Zhang, Cun Zhang

**Affiliations:** State Key Laboratory of Cancer Biology, Biotechnology Center, School of Pharmacy, The Fourth Military Medical University, Xi’an 710032, China; Shuningwang@fmmu.edu.cn (S.W.); lixiaoju1235@fmmu.edu.cn (X.L.); chidorie@fmmu.edu.cn (W.Z.); gaoyuan321@fmmu.edu.cn (Y.G.); justy1985@126.com (K.Z.); haosuq@fmmu.edu.cn (Q.H.); liweina@fmmu.edu.cn (W.L.); w1993@fmmu.edu.cn (Z.W.); limeng@fmmu.edu.cn (M.L.); zhangw90@fmmu.edu.cn (W.Z.); zhangyqh@fmmu.edu.cn (Y.Z.)

**Keywords:** genes, estrogen receptor alpha (ERα), breast cancer, RNA-seq, differentially expressed genes (DEGs), PPI networks

## Abstract

Estrogen receptor alpha (ERα), which has been detected in over 70% of breast cancer cases, is a driving factor for breast cancer growth. For investigating the underlying genes and networks regulated by ERα in breast cancer, RNA-seq was performed between ERα transgenic MDA-MB-231 cells and wild type MDA-MB-231 cells. A total of 267 differentially expressed genes (DEGs) were identified. Then bioinformatics analyses were performed to illustrate the mechanism of ERα. Besides, by comparison of RNA-seq data obtained from MDA-MB-231 cells and microarray dataset obtained from estrogen (E2) stimulated MCF-7 cells, an overlap of 126 DEGs was screened. The expression level of ERα was negatively associated with metastasis and EMT in breast cancer. We further verified that ERα might inhibit metastasis by regulating of VCL and TNFRSF12A, and suppress EMT by the regulating of JUNB and ID3. And the relationship between ERα and these genes were validated by RT-PCR and correlation analysis based on TCGA database. By PPI network analysis, we identified TOP5 hub genes, FOS, SP1, CDKN1A, CALCR and JUNB, which were involved in cell proliferation and invasion. Taken together, the whole-genome insights carried in this work can help fully understanding biological roles of ERα in breast cancer.

## 1. Introduction

Breast cancer is a heterogeneous cancer that remains to be the most common malignancy in females [1]. In the clinic, about 70% of breast cancers are estrogen receptor alpha (ERα) positive [1]. ERα, which is encoded by ESR1, has been recognized as a nuclear hormone receptor. It can be activated by directly interacting with estrogen [2]. ERα contains two activation domains, AF-1 and AF-2. AF-1 is activated by phosphorylation, while AF-2 can be activated in a ligand-dependent manner. These two transcription domains play a crucial role in ERα activation [3]. 

ERα can bind with estrogen and then lead to the synthesis of proteins which drive cell proliferation and survival. It has been regarded as the most frequently applied clinical biomarker of endocrine therapy for ERα-positive breast cancer [4]. However, ERα loss and ESR1 mutation have been detected in metastatic relapse of ERα-positive breast cancer [5,6]. Meanwhile, our previous research showed that ERα inhibited breast cancer metastasis through suppressing cell amoeboid-like movement [7]. Consequently, ERα acted as a main driver for cell proliferation and growth, while it was also an inhibitor for metastasis in breast cancer. These researches indicate the multiple roles of ERα and so it is necessary to get a deep insight on the mechanisms of ERα at the whole genomic level.

RNA sequencing (RNA-seq) is widely used to get whole-genome information in biological samples such as cells and tissues [8]. By the high-throughput capability, RNA-seq can identify genomic information with high sensitivity. Based on the transcriptional information, the subsequent bioinformatics analysis including gene ontology (GO) annotation and pathway analysis of (DEGs) could reveal functional clusters that genes are enriched and indicate the underlying pathways that genes are function in [9]. A number of studies have identified DEGs which can be considered as potential markers for diagnosis, prognosis and therapy [8,10,11,12]. 

In this article, in order to define the expression profiles and the roles of ERα in breast cancer, RNA-seq between ERα transgenic MDA-MB-231 and wide type MDA-MB-231 cells was carried. Meanwhile, according to the microarray data obtained from the Gene Expression Omnibus (GEO) database, which was performed in estrogen stimulated MCF-7 cells, DEGs between the estrogen stimulated and control groups were verified. Then, the common DEGs targeted by ERα in MDA-MB-231 and MCF-7 cell lines were identified. Furthermore, bioinformatics analysis on biological processes, pathway, GO function, and the protein-protein interaction (PPI) network of the DEGs were taken together to verify the function of ERα. At last, combining with the TCGA database, the correlation between ERα and target genes were verified and the effects of ERα were finally illustrated in breast cancer. This study was helpful for comprehensively understanding the effects of ERα in breast cancer.

## 2. Results

### 2.1. Identification of DEGs between ERα Transgenic MDA-MB-231 and Wild Type MDA-MB-231 Cells

**Figure 1:**
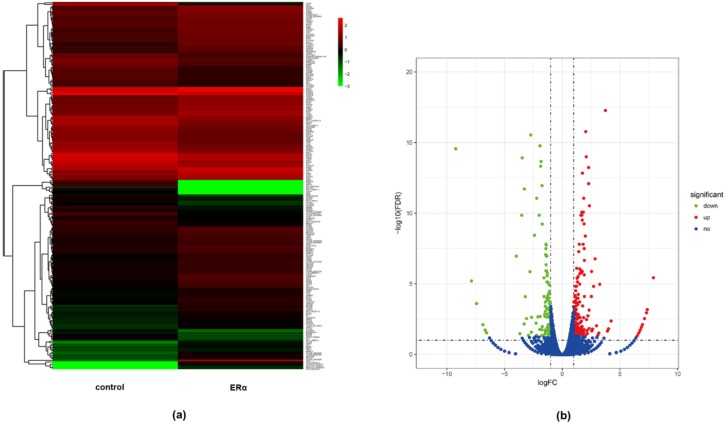
Identification of differentially expressed genes (DEGs) between ERα transgenic MDA-MB-231 and wild type MDA-MB-231 cells. (**a**) Hierarchical cluster of differential expression levels between ERα transgenic MDA-MB-231 cells and wild type MDA-MB-231 cells. The color scale represents log10 expression values, red indicates the high expression level, and the green refers to the low expression level. (**b**) The scatter plot of DEGs. Each point represents a gene. Red points represent up-regulated genes. Green points represent down-regulated genes. Blue points refer to genes without differential expression.

In order to identify genes regulated by ERα, ERα transgenic MDA-MB-231 cells were constructed and the real-time PCR and western-blot were used to verify ERα expression level. The results illustrated the high transcription and protein levels of ERα in ERα transgenic MDA-MB-231 cells (Figure S1). Then, the global gene expression analysis was performed between ERα transgenic MDA-MB-231 cells and wild type cells. A heat map of DEGs was constructed and expression changes of genes were showed by the hierarchical cluster analysis (Figure 1a). A total of 267 differentially expressed genes (DEGs) were identified with a criteria of *p* value < 0.05 and fold change (FC) ≥ 2 (Table S1). Furthermore, the volcano plot showed that among these DEGs, 163 genes were up-regulated and 104 genes were down-regulated (Figure 1b). 

### 2.2. Validation of Gene Expression Data by Real-Time PCR

**Figure 2:**
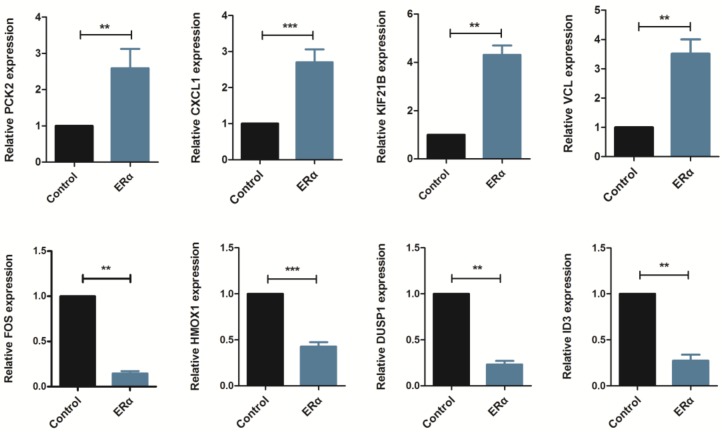
Validation by RT-PCR of eight randomly selected differentially expressed genes (DEGs). The *x*-axis denotes two groups, control (wild type MDA-MB-231) and ERα transgenic MDA-MB-231 cells. The *y*-axis refers to the relative expression level for each gene, with the mean ± SD of three replicates.

To validate the results of the RNA-seq, Real-Time PCR was carried out for randomly selected eight DEGs (PCK2, CXCL1, KIF21B, VCL, FOS, HMOX1, DUSP1 and ID3). The higher expression levels of PCK2, CXCL1, KIF21B and VCL were detected in ERα transgenic MDA-MB-231 cells than wild type cells. While FOS, HMOX1, DUSP1 and ID3 were identified with lower expression levels in ERα transgenic MDA-MB-231 cells compared with wild type cells (Figure 2). As a result, the expression differences obtained by RT-PCR were consistent with the results of the RNA-seq transcriptional analysis (Table 1). 

### 2.3. GO Enrichment of DEGs

**Figure 3:**
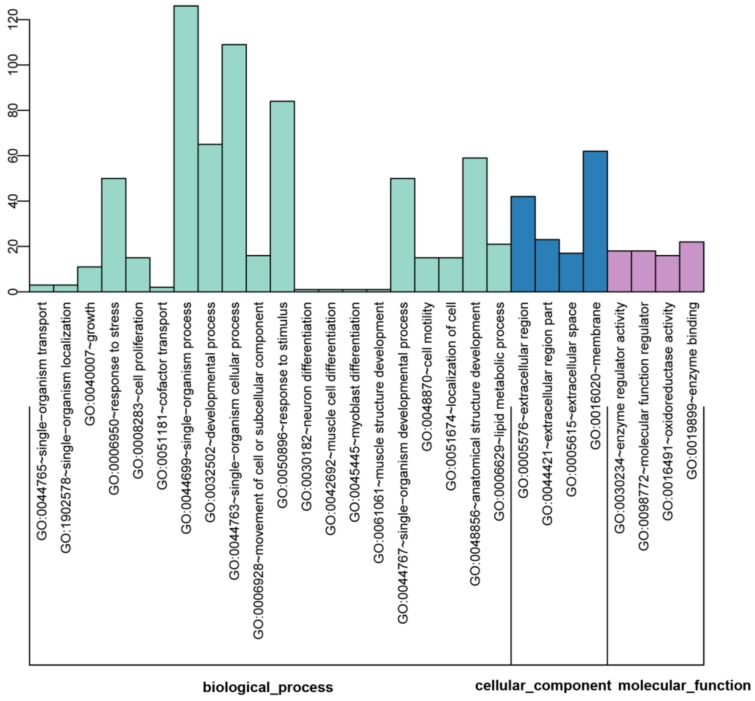
Functional enrichment of differentially expressed genes (DEGs) based on gene ontology (GO) categorization, including significantly enriched terms of biological process (BP), cellular component (CC) and molecular function (MF). The *x*-axis shows the ID and its corresponding category. The *y*-axis shows the number of DEGs enriched in each term.

The GO analysis was used to map the DEGs to specific functional categories. The DAVID database was performed to analyze the predominant functional themes on the GO hierarchy. 267 DEGs were supplied to GO analysis. The significantly enriched GO terms of DEGs between ERα transgenic MDA-MB-231 and wild-type cells were illustrated in Figure 3. 

In the biological process (BP) analysis, most of the DEGs mapped on “single-organism process”, “single-organism cellular process”, “response to stimulus”, “developmental process”, “anatomical structure development”, “response to stress”, “single-organism developmental process”, “lipid metabolic process”, “movement of cell or subcellular component”, “cell proliferation”, “cell motility” and “localization of cell”. In the cellular components (CC) analysis, the significantly enriched terms were “extracellular region”, “extracellular region part”, “extracellular space” and “membrane”. In the molecular function (MF) category, “enzyme regulator activity”, “molecular function regulator”, “oxidoreductase activity” and “enzyme binding” were the significantly mapped terms (Figure 3). 

### 2.4. Pathway Analysis of DEGs

**Figure 4:**
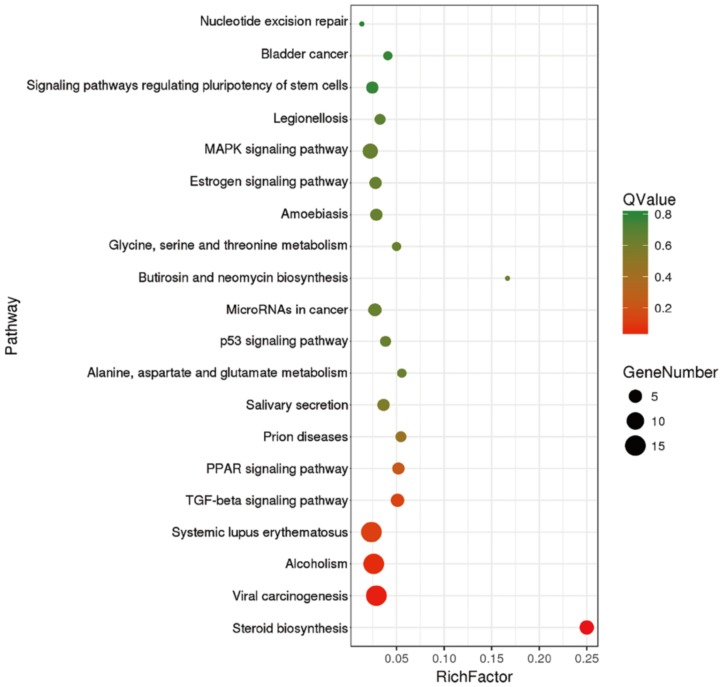
TOP20 significantly enriched KEGG pathways of ERα regulated DEGs. Each point represents a specific KEGG signaling pathway, and the size of the point indicates the number of DEGs enriched in each pathway. Rich Factor refers to the enrichment level of DEGs enriched to the pathway. Q value, the closer to zero indicating the enrichment is more significant.

To further specify the direct correspondence of the pathways and better to clarify the biological insights of ERα, the Kyoto Encyclopedia of Genes and Genomes (KEGG) database, which could find frequent and significant enriched pathways, was performed. The results demonstrated that the DEGs were enriched in lots of signal pathways, of which the main enriched terms were “viral carcinogenesis”, “Alcoholism”, “Systemic lupus erythematosus”, “MAPK signaling pathway”, “steroid biosynthesis”, “TGF-beta signaling pathway”, “MicroRNAs in cancer”, “PPAR signaling pathway”, “Salivary secretion”, “Amoebiasis”, “Estrogen signaling pathway” and “signaling pathways regulating pluripotency of stem cells”(Figure 4).

**Figure 5:**
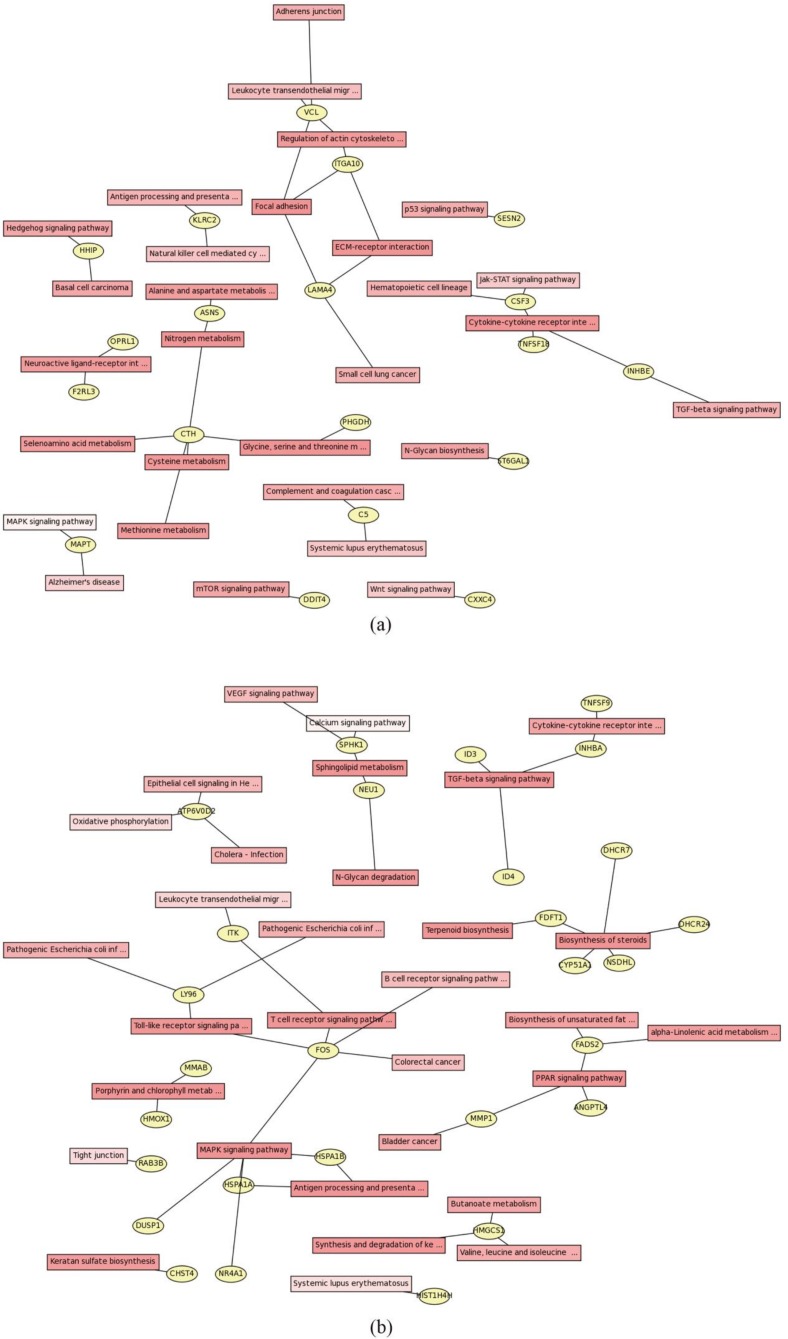
Pathway networks of differentially expressed genes. The yellow elliptical box means the DEGs and the quadrate box means the KEGG signaling pathway. The *p*-value from 0 to 1 is indicated in red to white. (**a**) KEGG pathway network of up regulated genes. (**b**) KEGG pathway network of down regulated genes.

Furthermore, we also analyzed the network of the signal pathways which DEGs participated in. Several core genes were identified in the downstream of ERα (Figure 5). As shown in Figure 5a, the up-regulated gene CTH enriched in nitrogen metabolism, glycine, serine and threonine metabolism, cysteine metabolism, methionine metabolism and selenoamino acid metabolism. Meanwhile, vinculin (VCL) enriched in adherens junction, leukocyte transendothelial migration and focal adhesion and it also modulated the ECM-receptor interaction through ITGA10. In the down-regulated genes, FOS acted as a core gene in the pathway network and it regulated T cell receptor signaling pathway, Toll-like receptor signaling pathway and MAPK signaling pathway (Figure 5b).

### 2.5. PPI Network Analysis

**Figure 6:**
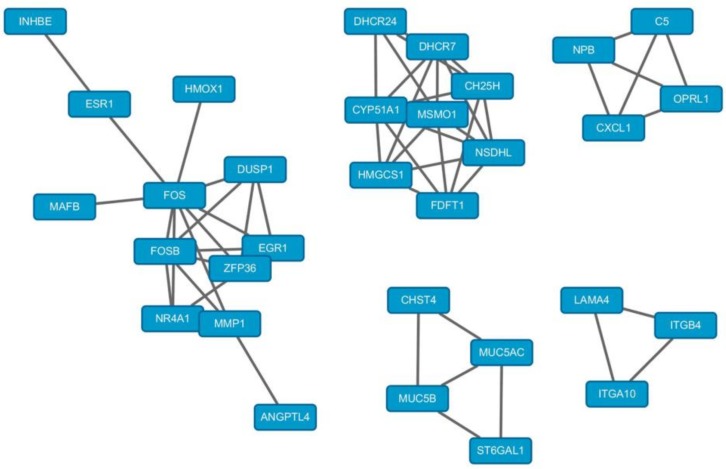
Protein-protein interaction networks of the DEGs. The network was composed of 31 nodes and 56 connections. In the network, the rectangle nodes represent the DEGs and the lines represent the predicted connections.

The analysis of protein–protein interaction (PPI) networks revealed that there were 31 genes related to each other among the DEGs. The total PPI network contained 31 nodes and 56 edges (Figure 6). Based on the information obtained from the STRING database, the top five hub nodes with higher degrees were screened. These hub genes were Fos proto-oncogene (FOS), cytochrome P450 family 51 subfamily A member 1 (CYP51A1), methylsterol monooxygenase 1 (MSMO1), nicotinamide adenine dinucleotide phosphate dependent steroid dehydrogenase-like (NSDHL) and FosB proto-oncogene (FOSB). Among these genes, FOS showed the highest degree, and it directly interacted with 10 genes, including ESR1, HMOX1, DUSP1, EGR1, ZFP36, MMP1, FOSB, NR4A1, ANGPTL4 and MAFB. These results indicated that FOS was an important gene in the downstream of ERα and it might participate in different biological process by interacting with other genes. 

### 2.6. Comparison of the DEGs Identified in ERα Transgenic MDA-MB-231 versus E2 Stimulated MCF-7 Cells

**Figure 7:**
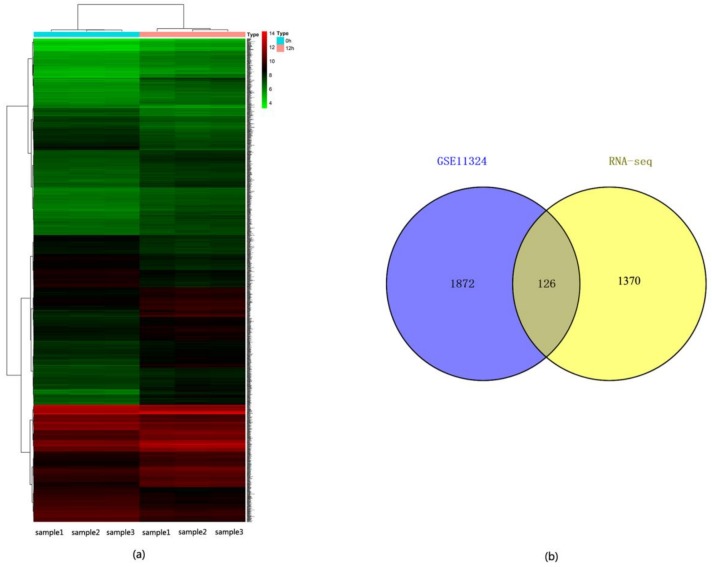
Analysis of differentially expressed genes (DEGs) for dataset GSE11324. (**a**) Heatmap showing the hierarchical cluster of differential expression levels between 12 h group of E2 stimulated MCF-7 cells and 0 h group. Gene expressed in different level is indicated in different colors. Red indicates the high expression level, and the green refers to the low expression level. (**b**) The overlapped DEGs between ERα transgenic MDA-MB-231 cells and E2 stimulated MCF-7 cells.

For further understanding the role of ERα in breast cancer, gene expression profile in E2 stimulated MCF-7 cells and control MCF-7 cells (accession no. GSE11324) was downloaded from the Gene Expression Omnibus (GEO) database. Groups in which with E2 stimulating of 12 h and 0 h were selected for analysis, and hierarchical clustering indicated that the DEGs were detected by estrogen treatment (Figure 7a). A total of 1998 DEGs were identified with at least 1.5-fold change and the *p* value was less than 0.05 (Table S2). Compared with 1496 DEGs obtained from our RNA-seq data by the same criteria (Table S3), 126 overlapped genes were found in ERα transgenic MDA-MB-231 cells and E2-stimulated MCF-7 cells (Figure 7b, Table S4).

**Figure 8:**
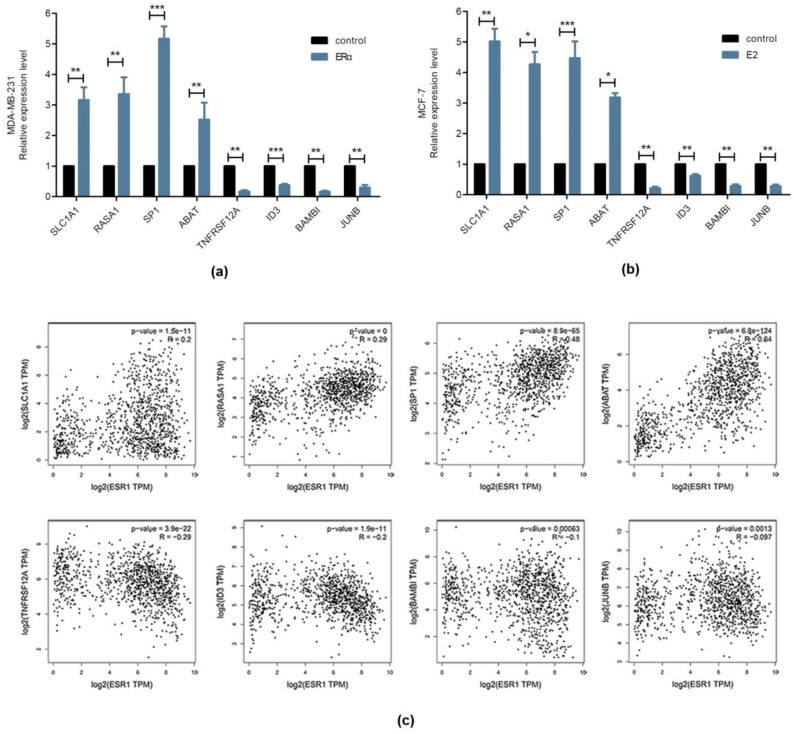
Verification expression level of eight overlapped DEGs in breast cancer cell lines. (**a**) Real-time PCR detecting the transcription levels of eight overlapped DEGs on MDA-MB-231 cell line by gain ERα. The results were normalized to GAPDH, with the mean ± SD of three replicates. (**b**) MCF-7 cells were treated with estrogen (E2) and subjected to quantitative reverse transcriptase PCR (qRT-PCR) assay (*n* = 3). (**c**) Verification common genes expression by Pearson correlation analysis based on TCGA breast tumor dataset.

To validate the expression level of common genes identified between ERα transgenic MDA-MB-231 and E2-stimulated MCF-7 cells. Real-Time PCR for eight randomly selected genes, including SLC1A1, RASA1, SP1, ABAT, TNFRSF12A, ID3, BAMBI and JUNB was conducted. As shown in Figure 8a,b, the results indicated that the expression of SLC1A1, RASA1, SP1 and ABAT was up-regulated, while TNFRSF12A, ID3, BAMBI and JUNB was down-regulated in ERα containing 231 cells and E2-stimulated MCF-7 cells. These results were generally consistent with the expression changes detected by RNA-seq and microarray dataset (Table 2). To further verify the relationship between these genes and ERα, TCGA dataset with a total of 1070 breast tumor patients were included in our analysis. Through analysis, we found that SLC1A1, RASA1, SP1 and ABAT showed positive correlation with ESR1, while TNFRSF12A, ID3, BAMBI and JUNB showed negative correlation with ESR1 (Figure 8c). 

For further investigating the consistency of the function of ERα in different breast cancer subtype, we downloaded the RNA-seq data of breast invasive carcinoma patients cohort of estrogen receptor positive and negative from The Cancer Genome Atlas (TCGA) database. Then, 12570 DEGs were identified with fold change ≥ 1.5 (Table S5). Subsequently, the overlapped 126 DEGs were compared with 12570 DEGs that were identified between a cohort of estrogen receptor positive and negative patients. As a result, 48 common genes were verified (Figure S2). These results indicated that the DEGs identified from ERα transgenic MDA-MB-231 and E2-stimulated MCF-7 cell lines shared an overlap of genes with that observed from the patients cohort of estrogen receptor positive and negative.

Additionally, in order to identify the DEGs with ERα binding sites, we also compared the 126 overlapped DEGs with 737 genes that were detected with ERα binding sites by Chip-PET [13] and 578 genes which were identified with ERα binding sites by Chip-DSL respectively [14]. As Figure S3 showing, 12 genes including CYP1A1, ABAT, ELF3, GREB1, FOS, SLC1A1, BCAS3, RAB31, C6orf141, PKIB, ELF3 and GREB1 were identified with ERα binding sites. These results suggested that the 12 genes mentioned above might be directly regulated by ERα. 

### 2.7. Bioinformatics Analysis of Common Genes Detected in ERα Transgenic 231 versus E2 Stimulated MCF-7 Cells

**Figure 9:**
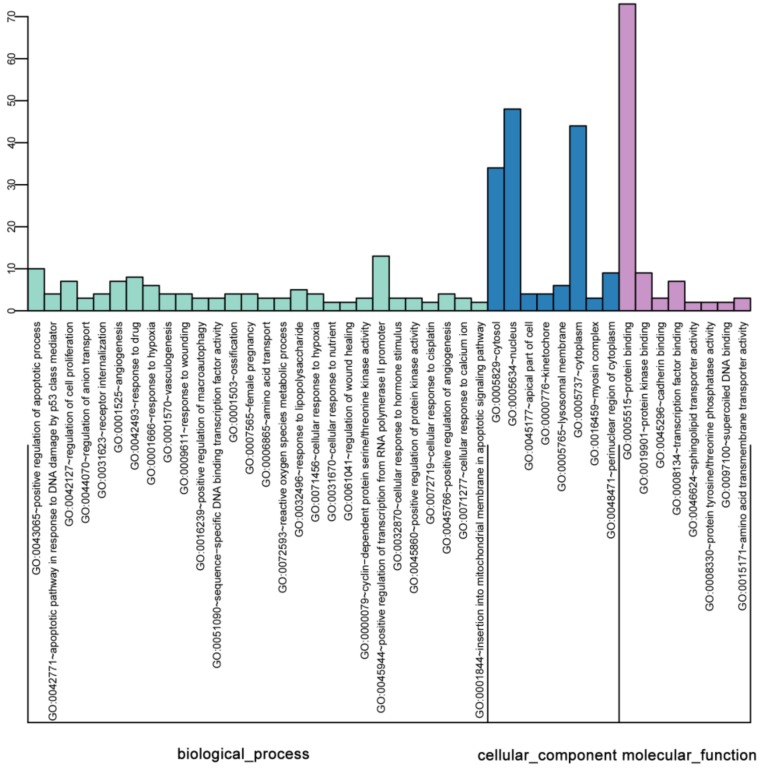
Functional enrichment of overlapped DEGs between ERα transgenic MDA-MB-231 and estrogen stimulated MCF-7 cells based on gene ontology (GO) categorization, including significantly enriched terms of biological process (BP), cellular component (CC) and molecular function (MF). The *X* axis shows the term ID and its corresponding category. The *Y* axis shows the number of DEGs enriched in each term.

For deeply understanding function of common genes detected in ERα transgenic 231 versus E2 stimulated MCF-7 cells, bioinformatics analysis including GO enrichment and PPI network analysis were performed. The most significantly enriched GO terms of common genes were illustrated in Figure 9. Screened biological processes of overlapped DEGs were important for the understanding of ERα in breast cancer. After analysis, we found that overlapped genes were closely related to biological processes associated with “positive regulation of apoptotic process”, “intrinsic apoptotic signaling pathway in response to DNA damage by p53 class mediator”, “regulation of cell proliferation”, “regulation of anion transport”, “receptor internalization”, “angiogenesis”, “response to drug”, “response to hypoxia”, “vasculogenesis” and “response to wounding”. For the cellular component category, the main enriched terms were “cytosol”, “nucleus” and “cytoplasm”. For the molecular function, “protein binding” was the main enriched term. 

**Figure 10:**
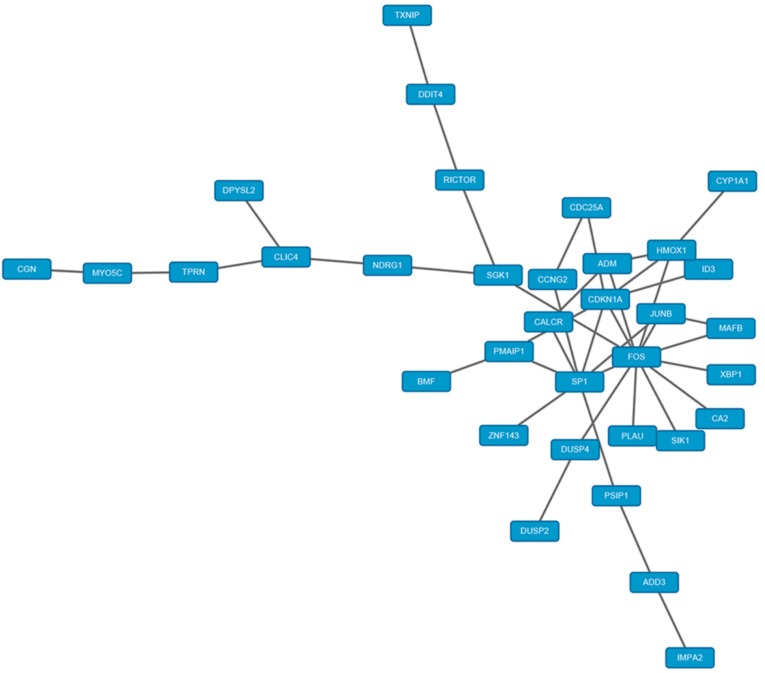
Protein-protein interaction networks of the overlapped DEGs between ERα transgenic MDA-MB-231 and E2 stimulated MCF-7 cells. In the network, the rectangle nodes represent the DEGs and the lines represent the predicted connections.

By the protein-protein interaction network analysis, we obtained a total of 41 protein-protein interaction pairs with the combined score > 0.7. Figure 10 shows the PPI network visualized by cytoscape. Based on the network, the top five hub genes were identified. These hub genes included Fos proto-oncogene (FOS), Sp1 transcription factor (SP1), cyclin dependent kinase inhibitor 1A (CDKN1A), Calcitonin receptor (CALCR) and AP-1 transcription factor subunit (JUNB). Among these genes, FOS also showed the highest node degree, and it directly interacted with 12 genes including JUNB, HMOX1, SP1, PLAU, MAFB, ADM, SIK1, CDKN1A, XBP1, DUSP4, SGK1 and CA2. These results revealed that FOS might play a crucial role in ERα containing breast cancer. 

## 3. Discussion

ERα, as a member of nuclear hormone receptor family, mediates a broad range of biological functions [15]. Moreover, ERα-positive tumors constitute the largest proportion in breast cancer [16,17]. Therefore, understanding the molecular mechanism of ERα in breast cancer, especially on the whole-genome level is of vital importance. In the present study, RNA-seq was used to illustrate the roles of ERα in breast cancer. Through analysis, 163 up-regulated and 104 down-regulated DEGs were identified in ERα transgenic MDA-MB-231 cells compared with wide type cells. Then the bioinformatics analyses, including KEGG pathway analysis, GO enrichment analysis and PPI network construction were taken together to verify ERα regulating genes and their functions.

A number of studies identified gene expression changes and binding sites for ERα under estrogen (E2) stimulation, and these researches have all been carried in ER-positive breast cancer cell line MCF-7 [13,18,19]. Interestingly, it is reported that by comparing the ERα binding sites between 231 ERα+ cells and E2-stimulated MCF-7 cells, an overlap of 56% were detected, suggesting that ERα in 231 ERα+ cells shared common sites with that observed in MCF-7 cells [20]. Hence, there are considerable similarities for the regulation of ERα in different human breast cancer cell lines. In order to fully understanding the biological functions of ERα, we also compared our RNA-seq data with microarray dataset obtained from MCF-7. An overlap of 126 DEGs between ERα transgenic MDA-MB-231 and E2-stimulated MCF-7 cells were screened. And the overlapped genes were mainly enriched in the biology process associated with breast tumor progression. It is well known that ERα regulates genes by different ways. It can directly bind to target genes at estrogen response elements (EREs) [21]. Meanwhile, it also can indirectly bind with some transcription factors [20]. In the present study, among the 126 overlapped DEGs, 12 genes were verified with ER binding sites by Chip-PET and Chip-DSL respectively. So, the other genes might be mediated via indirectly way.

By the bioinformatics analysis, we found that many DEGs were enriched in cell growth and proliferation, such as specificity protein 1 (SP1) and high-mobility group box 2 (HMGB2). SP1 is a transcription factor that has been detected in many tissues and regulates several cellular processes [22]. Furthermore, by regulating genes associated with tumor growth and proliferation, SP1 has been reported to promote tumorigenesis and progression [23]. And the binding sites between SP1 and ERα have been confirmed [18]. HMGB2 belongs to the high-mobility group box (HMGB) protein family, which can mediate DNA binding and bending functions [24]. Recent study indicated that HMGB2 was a positive regulator of proliferation and glycolysis in breast cancer cells [25]. In our data, these two genes were all up-regulated by ERα. This is consistent with the previous research, which revealed that ERα is a primary driver of growth in ERα positive breast cancers [21,26].

Beyond tumor growth, we also detected several DEGs enriched in metastasis, including vinculin (VCL) and Tumor necrosis factor (TNF) receptor superfamily member 12A (TNFRSF12A). Vinculin (VCL) is known as a cytoskeletal protein. It is involved in cell-extracellular matrix junctions and cell-cell junctions [27]. The expression of vinculin can inhibit tumor invasion, whereas loss of vinculin promotes cell motility and migration [28,29]. In this data, VCL was up-regulated by ERα, and the result was consistent with our previous research in which ERα inhibited amoeboid-like migration of breast cancer cells by up-regulating vinculin [7]. TNFRSF12A is a member of the TNF superfamily of receptors. Knockdown of TNFRSF12A can inhibit hepatocellular carcinoma cell proliferation and migration in vitro [30]. Besides, TNFRSF12A acts as a drive factor in bone metastasis of prostate cancer [31]. In the present study, we found that TNFRSF12A was down-regulated in ERα transgenic MDA-MB-231 cells and E2-stimulated MCF-7 cells (Table 2, Figure 8). The negative correlation between ERα and TNFRSF12A was confirmed by RT-PCR and a given set of TCGA expression data. In addition, based on gene expression and clinical information of 3951 patients with breast cancer, we analyzed the relationship between ERα or target genes expression and survival by the Kaplan-Meier plotter [32,33]. We found that high ERα expression is a protective factor for breast cancer survival, while high TNFRSF12A expression is a deleterious factor (Figure S4). Furthermore, less than 45.5% of the patients exhibiting high ERα expression showed high TNFRSF12A expression. In contrast, more than 54.5% of the patients exhibiting low ERα expression showed high TNFRSF12A expression. And this difference was significant with a *p* value less than 0.001 (Table S6). These results suggested that simultaneous ERα up-regulation and TNFRSF12A down-regulation were associated with increased breast cancer survival, and TNFRSF12A might be a potential therapeutic target for breast cancer.

Epithelial-to-Mesenchymal Transition (EMT) is a biological process and in which, epithelial cells lose cell-cell junctions to acquire an invasive mesenchymal phenotype. Recently abnormal reactivation of EMT has been detected in cancer [34]. It has been reported that ERα inhibits EMT by the regulation of Bmil, TBK1 and slug [35,36,37]. As we know, the transforming growth factor β (TGF-β) signaling pathway initiates EMT process by inducing loss of cell-cell adhesions [38,39]. In this study, two genes in TGFβ pathway were identified, including JunB proto-oncogene, AP-1 transcription factor subunit (JUNB) and inhibitors of differentiation 3 (ID3). JUNB belongs to activator protein-1 (AP-1) family. As an early response gene of TGF-β signaling pathway, the expression of JUNB plays a crucial role in cell-matrix adhesion of epithelial cell systems, and silence of JUNB can inhibit the dissolution of cell-cell junctions [40,41]. JUNB has been demonstrated as a driver in TGF-β induced EMT [40]. ID3 is a member of the family of helix-loop-helix (HLH) transcription factors. It can dimerize with other transcriptional regulators and directly bind to several transcription factors to inhibit their activity [42]. ID3 plays a role in TGFβ mediated cell migration in prostate cancer cells [43]. Meanwhile, it is required for breast cancer lung metastases [44]. Additionally, ID3 can mediate epithelial-mesenchymal plasticity via the TGF-β signaling [45]. These two genes were both down-regulated by ERα (Table 2, Figure 8). The relationship between ERα and these two genes was consistent with the RT-PCR and correlation analysis based on TCGA database (Figure 8c). Hence, we inferred that ERα might suppress EMT via TGF-β pathway by the down-regulating of JUNB and ID3.

Beyond TGF-β signaling pathway, our study also found that the overexpression of ERα can regulate several other pathways, including mitogen-activated protein kinases (MAPK) signaling pathway, pathway regulating pluripotency of stem cells and PPAR signaling pathway. MAPK signaling pathway, which contributes to tumor growth and progression, plays a critical role in the development of breast cancer [46]. In this study, we found that ERα can increase the transcription level of genes enriched in MAPK signaling pathway. This is consistent with recent researches which revealed that the overexpression of ERα can induce increased activated downstream molecules of MAPK pathway in breast cancer [47]. Regulating pluripotency of stem cells can improve the capability of self-renew and then promote the formation of cancer stem cells which participate in tumorigenesis, metastasis and relapse [48,49]. Moreover, it has been revealed that ERα may direct silencing of a number of cancer stem cell genes in breast cancer [50]. This is consistent with this study which suggested that ERα negatively regulated the DEGs enriched in regulating pluripotency of stem cells. Peroxisome proliferator-activated receptors (PPARs) are members of the nuclear hormone receptor superfamily. They are involved in several biological processes including proliferation and lipid metabolism, and play a significant role in cancer [51]. In this study, we found that ERα suppressed lipid metabolism by down-regulating genes involved in PPARs signaling pathway.

The PPI network constructed by overlapped DEGs identified five hub genes, including FOS, SP1, CDKN1A, CALCR and JUNB. Among these five genes, FOS showed the highest degree. FOS is a member of activator protein-1 (AP-1) family. It can dimerize with proteins of the JUN family, and then form AP-1 complex. FOS is involved in several biological processes, such as cell proliferation, differentiation, apoptosis, and transformation [52]. Furthermore, the deregulation of FOS might associate with tumor progression and oncogenic transformations [52]. As the core gene regulated by ERα, FOS might play a crucial role in ERα positive breast cancer.

It has been reported that endocrine therapy, including tamoxifen and an aromatase is widely used for ER positive breast cancer. Resistance to endocrine therapy is a major clinical problem [16]. Many studies focus on illustrating the mechanisms of tamoxifen resistance [53,54]. Elias et al. performed gene array analyses and explored gene expression changes between four unique tamoxifen-resistant (TamR) cell lines and the parental tamoxifen-sensitive MCF-7/S0.5 cell line [53]. By comparing the DEGs identified in our data with that detected by Elias et al., we found that six genes regulated by ERα also showed correlation with endocrine treatment responses. As Table S7 showing, INHBE, CHAC1, SLC1A4, NDRG1, MTHFD2 and RAB31 were all up-regulated by ERα in our data, and they were also detected with higher expression level in tamoxifen-sensitive cell lines. These results indicated that the overexpression of ERα might strengthen the sensitivity of endocrine treatment via the up-regulation of INHBE, CHAC1, SLC1A4, NDRG1, MTHFD2 and RAB31. Therefore, overexpression of the six genes mentioned above might improve endocrine treatment responses for resistant breast cancer cell lines. 

It is well known that MCF-7 cell line is luminal type and MDA-MB-231 cell line is basal-like/triple negative type. Simultaneously, our previous research showed that ectopic expression of ERα promoted proliferation of MDA-MB-231 cells in vitro and in vivo [7]. In addition, it has been reported that it is possible to converse basal-like breast cancers into a hormone receptor-positive state that conferred sensitivity to endocrine therapy in previously impervious tumors by genetic or pharmacological intervention with PDGF-CC activity in mouse models [55]. Collectively, these studies indicated that the different breast cancer subtype could be converted to one another and that we might develop new therapy especially for basal-like/triple negative type of breast cancer. Therefore, it is meaningful to transfer the wild type ERα into basal-like breast cancer to verify the genome wide changes, which may provide important information for unknown mechanisms that contribute to breast cancer subtype and sheds light on clinical therapeutic for patients with basal-like breast cancers.

## 4. Materials and Methods

### 4.1. Cell Culture

Human breast cancer cell lines MDA-MB-231 and MCF-7 were obtained from the Type Culture Collection of the Chinese Academy of Sciences (Shanghai, China). The MDA-MB-231 cells were routinely cultured in Leibovitz’s L-15 Medium (HyClone, Logan, UT, USA) containing 10% foetal calf serum (HyClone, Logan, UT, USA), and in incubator of 37 °C, without CO_2_. MCF-7 cells were cultured in DMEM Medium (HyClone) containing 10% foetal calf serum, and in incubator of 37 °C.

### 4.2. Construction of ERα Transgenic MDA-MB-231Cell Line

The MDA-MB-231 cells were seeded in six-well plate at a density of 2 × 10^5^ cells/well. The cells were cultured overnight in incubator of 37 °C, without CO_2_. The pcDNA3.1 (−)-ERα plasmid was retained in our laboratory. The plasmid was transfected into MDA-MB-231 cell line by Lipofectamine 3000 (Invitrogen, Carlsbad, CA, USA) following the manufacturer’s instructions. 

### 4.3. Western-Blotting

Cell samples were pelleted, washed with PBS and lysed in a lysis buffer (Beyotime, Shanghai, China). Protein was estimated using Bradford reagent and 30 μg of proteins was loaded on SDS-PAGE. Then the proteins were transferred to polyvinylidene difluoride membranes (Millipore, Billerica, MA, USA) after SDS-PAGE using a Bio-Rad Semi-Dry electrophoretic cell. The PVDF membrane was incubated using GAPDH antibody (CW0101, CWBIOTECH), ERα antibody (ab32063, Abcam, Cambridge, England) and horseradish peroxidase (HRP)-conjugated IgG antibody. Enhanced chemiluminescence (Thermo Scientific, Rockford, IL, USA) for HRP was used for immunoreactive protein visualization. GAPDH was used as internal control.

### 4.4. RNA-Seq Analysis

Total RNAs for ERα transgenic MDA-MB-231 and wide type MDA-MB-231 cells were extracted by TRIzol reagent (Invitrogen, Carlsbad, CA, USA) according to the protocol. Following assessment of RNA-integrity, ERα transgenic and wide type MDA-MB-231 cells were used for RNA-seq. After the total RNA was extracted from the sample, the RNA was enriched with Oligo (dT) magnetic beads, then fragmentation buffer was added into the RNA to get the short fragment. Using the short fragment as template, the first chain of cDNA was synthesized by random-hexamer primers. The second strand of cDNA was synthesized by dNTPsNase H and DNA polymerase I. The cDNA was then purified by using QIAquick PCR kit (Qiagen, Hilden, Germany) and eluted with EB buffer. Then the whole library was constructed and sequenced by Illumina HiSeqTM 4000 (Illumina Inc., San Diego, CA, USA). The expression levels for each gene to the reads per kilobase of transcript per million fragments mapped (RPKM) were normalized to facilitate the comparison of transcripts between samples. The RNA-seq data have been uploaded to the NCBI Sequence Read Archive (SAR accession: SRP156856).

### 4.5. Identification of Differentially Expressed Genes (DEGs)

The computational detection method EdgeR (empirical analysis of digital gene expression data in R) was conducted to identify the differentially expressed genes between the ERα transgenic and wide type MDA-MB-231 cells [56]. And the reads per kilobase of transcript per million fragments mapped (RPKM) were then log2 transformed. The criteria of detecting DEGs were *p* < 0.05 and |log_2_FC| ≥ 1. 

### 4.6. Validation by RT-PCR

To validate the accuracy of the RNA-seq data, eight genes were randomly selected for validation of gene expression using quantitative RT-PCR. Total RNA (100 ng) was reverse transcribed into single stranded cDNAs using SuperScript III reverse transcriptase (Invitrogen, Carlsbad, CA, USA) and Oligo (dT) (Invitrogen, Carlsbad, CA, USA) in 20 μL reaction at 57 °C for 50 min, 85 °C for 5 min, 37 °C for 20 min. 2μL of cDNA was used for a subsequent 20 μL PCR amplification. RT-PCR was carried out using Prism Expression Assays 7500 (Applied BioSystems, Foster City, CA, USA). The reaction protocol contained 10 min at 94 °C, followed by 50 cycles of 15 s at 94 °C, 20 s at 60 °C, and 30 s at 72 °C. Relative expression level of genes was calculated by the 2^−∆∆Ct^ method. The primer sequences are provided in Table S8. The reactions were performed in triplicate. GAPDH was used as the internal control.

### 4.7. GO Enrichment and KEGG Pathway Analysis

To obtain functional annotation of genes regulated by ERα, gene ontology (GO) analysis were performed using the database for annotation, visualization and integrated discovery (DAVID) version 6.8 (http://david.abcc. ncifcrf.gov/) [57]. By this way, the biological process, cellular component, molecule function and enriched pathway of target genes can be defined graphically. Pathway analysis was carried by the Kyoto Encyclopedia of Genes and Genomes (KEGG) database (https://www.kegg.jp) [58]. The gene-pathway network was conducted using MAS (molecule annotation system) 3.0 platform (http://bioinfo.capitalbio.com/mas3/) with the default parameters. The up and down-regulated genes were separated. Enriched items with *p* value <0.05 was considered to be statistically significant.

### 4.8. Construction of Protein-Protein Interaction (PPI) Networks 

A list of DEGs was supplied to the online program Search Tool for the Retrieval of Interacting Genes (STRING) version 10.5 (https://string-db.org/) [59]. The programs were used to predict potential protein-protein interactions that may be common or unique to the ERα transgenic MDA-MB-231 and wild type cells, in order to provide a visual output of the molecular actions taking place within the generated networks. The associations among DEGs with a combined score >0.7 were identified by STRING. Then the PPI network was constructed and visualized using Cytoscape version 3.6.1 (http://www.cytoscape.org/) [60]. DEGs were clustered into different modules based on the combined score.

### 4.9. Microarray Data Analysis

The microarray data were obtained from Gene Expression Omnibus (GEO) with the accession number GSE11324 [61]. Gene expression profiles were screened upon 100 nM estrogen stimulation at different time point of 0 h, 3 h, 6 h and 12 h. Each group was made in triplicate. The raw dataset were quantificated by an Affymetrix GeneChip^®^ Human Genome U133 Plus 2.0 Array (Affymetrix, CA, USA), and the DEGs were identified between 12 h and 0 h by limma with R package [62]. Thresholds of fold change ≥ 1.5 and *p* value < 0.05 were used for the screening of DEGs.

### 4.10. Validation Overlapped Genes by RT-PCT and The Cancer Genome Atlas (TCGA) Analysis

RT-PCR was performed follow the procedure mentioned before, the primers of these eight randomly selected overlapped genes were listed in Table S8. The validation was carried in two breast cancer cell lines, including ERα transgenic and wide type MDA-MB-231 cells, and 100 nM Estrogen (Sigma, St. Louis)-stimulated and wide type MCF-7 cells. For further verifying the relationship between ERα and target genes at clinical level, GEPIA (http://gepia.cancer-pku.cn/index.html), which is an interactive web server for analyzing the RNA sequencing expression data of 9736 tumors and 8587 normal samples from the TCGA and the GTEx projects, was proceed using a standard processing pipeline [63]. Pearson correlation analyses between ERα and target genes were performed based on the TCGA Breast invasive carcinoma (BRCA) database. And we used the non-log scale for calculation and the log-scale axis for visualization. A *p* value < 0.05 was considered to be statistically significant.

### 4.11. Verification Genes with ERα Binding Sites

The list of 1234 high confidence ER binding site ChIP-PET clusters was downloaded from Lin et al. [13]. Then, the location information of 1234 ER binding sites was uploaded to the UCSC Genome Browser (http://genome.ucsc.edu) to verify target genes with these binding sites. In addition, another list of 578 genes with ER binding sites was obtained from Kwon et al. [14]. Then, the common genes between DEGs identified in this study and genes with ERα binding sites were explored and illustrated by the online program Venny 2.1 (http://bioinfogp.cnb.csic.es/tools/ venny/).

### 4.12. Identification of DEGs between ER Positive and Negative Breast Cancer Patients

Transcriptome profiling data of RNA-seq and corresponding clinical data were downloaded from the TCGA database (The Cancer Genome Atlas, https://cancergenome.nih.gov/). The cohort of estrogen receptor negative and positive breast cancer was classified by clinical data of each patient. 781 estrogen receptor positive breast cancer samples and 227 estrogen receptor negative breast cancer samples were involved in the study. EdgeR (Robinson, Melbourne, Australia) was also used to identify the DEGs between ER positive and negative breast cancer patients [56]. The criteria was *p* < 0.05 and fold change (FC) ≥ 1.5.

### 4.13. Kaplan–Meier Plotter Analysis

Survival analysis was carried on the Kaplan–Meier plotter website (www.kmplot.com), an online database which can identify the effect of specific gene on the prognosis of breast cancer, ovarian cancer, lung cancer and gastric cancer patients [32,33]. Briefly, the gene names were supplied into the database, and the 3951 breast cancer patients included in the analysis were divided into two cohorts by the median expression level of ERα and TNFRSF12A in breast cancer. Relapse-free survival (RFS) of patients in different cohorts was analyzed by Kaplan-Meier plots. The hazard ratio (HR) and log-rank *p* value were determined using the database and displayed. The expression levels of ERα and TNFRSF12A were also obtained by the survival analysis for further exploring the relationship between ERα and TNFRSF12A. 

### 4.14. Statistical Analysis

Statistical analysis was carried using the SPSS (SPSS 16.0, SSPS Inc., Chicago, IL, USA). A value of *p* < 0.05 was considered statistically significant. The statistical tests were two-sided. The data are shown as the mean ± SD. * *p* < 0.05, ** *p* < 0.01, *** *p* < 0.001.

## 5. Conclusions

In conclusion, a total of 267 DEGs with at least 2-fold change were identified in ERα transgenic MDA-MB-231 cells compared with wild type MDA-MB-231 cells. The GO enrichment, KEGG pathway, PPI network analysis were taken together to illustrate the roles of ERα in breast cancer. For further understanding the roles of ERα, we compared our RNA-seq data with microarray dataset obtained from ER-positive breast cancer cell line MCF-7. 126 overlapped DEGs were verified by cross analysis of gene expression changes between ERα transgenic MDA-MB-231 and E2-stimulated MCF-7 cells. Furthermore, we verified that ERα might inhibit metastasis through regulating of VCL and TNFRSF12A, and suppress EMT by the down regulating of BAMBI and ID3. This work might provide a genome-wide insight on the roles of ERα, which would give useful information on basic research and treatment of breast cancer.

## Figures and Tables

**Table 1:** Expression changes of genes detected by RNA-seq data.

Genes	Gene ID	RNA-Seq	
		Fold Change	Significant
ESR1	2099	1053	Up
PCK2	5106	2.57	Up
CXCL1	2919	2.29	Up
KIF21B	23046	7	Up
VCL	7414	6.63	Up
FOS	2353	0.02	Down
HMOX1	3162	0.41	Down
DUSP1	1843	0.3	Down
ID3	3399	0.45	Down

**Table 2:** Expression changes of eight overlapped DEGs detected by RNA-seq and microarray data.

Genes	RNA-Seq		Microarray Data		Significance
	Fold Change	*p*-Value	Fold Change		*p*-Value
SLC1A1	1.503	0.0406	1.878	2.18 × 10^−6^	up
RASA1	1.71	0.000281	1.879	2.89 × 10^−6^	up
SP1	1.603	0.005176	1.801	6.38 × 10^−6^	up
ABAT	2.545	0.005576	1.857	4.42 × 10^−8^	up
TNFRSF12A	0.67	0.0029	0.65	1.84 × 10^−5^	down
ID3	0.446	3.05 × 10^−08^	0.5	8.36 × 10^−8^	down
BAMBI	0.42	7.05 × 10^−07^	0.491	1.65 × 10^−8^	down
JUNB	0.65	0.003262	0.55	5.20 × 10^−7^	down

## Supplementary Materials

The following are available online at http://www.mdpi.com/1420-3049/23/10/2543/s1, Figure S1: Validation ERα expression level in ERα transgenic MDA-MB-231 cells compared with wild type MDA-MB-231 cells. (a) Real-time PCR detecting the transcription levels of ERα in ERα transgenic MDA-MB-231 cells and wild type 231 cells. (b) Western-blot was conducted to detect the protein levels of ERα in ERα transgenic MDA-MB-231 cells and wild type 231 cells. Figure S2: Venn diagram illustrating the common genes between the DEGs identified from ERα transgenic MDA-MB-231 and E2-stimulated MCF-7 cell lines with that observed in patients cohort of estrogen receptor positive and negative. Figure S3: Verification the common genes between ERα regulated genes and genes with ERα binding sites. (a) Venn diagram showing the common genes between ERα regulated genes and ERα Chip-PET data, and common genes are also shown. (b) The common genes between ERα regulated genes and ERα Chip-DSL data, and common genes are listed. Figure S4: Kaplan-Meier survival curves for relapse-free survival (RFS) in breast cancer patients with high ERα expression or low ERα expression and high TNFRSF12A expression or low TNFRSF12A expression. Table S1: DEGs between ERα transgenic MDA-MB-231 cells and wild type MDA-MB-231 cells with the |log2FC| ≥ 1. Table S2: The list of DEGs in E2 stimulated MCF-7 cells according to microarray dataset with fold change ≥ 1.5. Table S3: The list of DEGs between ERα transgenic MDA-MB-231 cells and wild type 231 cells with fold change ≥ 1.5. Table S4: The list of overlapped genes between ERα transgenic MDA-MB-231 cells and E2-stimulated MCF-7 cells. Table S5: The list of DEGs between patients cohort of estrogen receptor positive and negative. Table S6: Significant reduction in the rate of TNFRSF12A up regulation in the ERα positive breast cancer samples. Chi-square test was used in this analysis. Table S7: Expression changes of seven DEGs detected by RNA-seq in our data and tamoxifen-sensitive cell lines vs resistant cell lines. Table S8: Primer information of differentially expressed genes used for Real-Time PCR.
