# Supplementary material for: Genome-Wide Investigation of Genes Regulated by ERα in Breast Cancer Cells
Source: Molecules. 2018 Oct 5;23(10):2543. doi: 10.3390/molecules23102543 (PMC6222792; doi:10.3390/molecules23102543)
Supplement: Supplementary file 1 [file molecules-23-02543-s001.zip › supplementary/Table S6.docx]

**Table S6：Significant reduction in the rate of TNFRSF12A up regulation in the ERα+ breast cancer samples**

|  |  | ESR1 | |  | *P* value |
| --- | --- | --- | --- | --- | --- |
|  |  | low | high | Total |  |
| TNFRSF12A | low | 902 | 1077 | 1979 |  |
|  | high | 1074 | 898 | 1972 |  |
|  | Total | 1976 | 1975 | 3951 | P<0.001 |

Chi-square test was used in this analysis.
