# Supplementary material for: Genome-Wide Investigation of Genes Regulated by ERα in Breast Cancer Cells
Source: Molecules. 2018 Oct 5;23(10):2543. doi: 10.3390/molecules23102543 (PMC6222792; doi:10.3390/molecules23102543)
Supplement: Supplementary file 1 [file molecules-23-02543-s001.zip › supplementary/Table S7.docx]

**Table S7.** Expression changes of six DEGs detected by RNA-seq in our data and tamoxifen-sensitive cell lines vs resistant cell lines.

| **Genes** | **RNA-seq** | | | **Elias et al** | |
| --- | --- | --- | --- | --- | --- |
|  | **Fold Change** | **P-value** | **Fold Change** | | **P-value** |
| INHBE | 22 | 0.000253 | 4.556734 | | 3.85E-05 |
| CHAC1 | 3.709016 | 9.41E-15 | 4.175648 | | 1.43E-04 |
| SLC1A4 | 2.641026 | 1.03E-05 | 6.381962 | | 5.07E-06 |
| NDRG1 | 1.637677 | 0.000313 | 5.322116 | | 4.48E-06 |
| MTHFD2 | 1.589134 | 0.00333 | 4.836623 | | 1.28E-05 |
| RAB31 | 1.52528 | 0.002894 | 43.83466 | | 1.52E-06 |
