# Supplementary material for: Genome-Wide Investigation of Genes Regulated by ERα in Breast Cancer Cells
Source: Molecules. 2018 Oct 5;23(10):2543. doi: 10.3390/molecules23102543 (PMC6222792; doi:10.3390/molecules23102543)
Supplement: Supplementary file 1 [file molecules-23-02543-s001.zip › supplementary/Table S8.docx]

**Table S8：Primer information of differentially expressed genes used for real-time PCR**

| Gene name | Primer sequences(5’-3’) | Tm (∘C) | Product size |
| --- | --- | --- | --- |
| ESR1 | TGCAATGGAAACCTGACGGA AAGTTCTCCACTCTCCCCCA | 59 | 191 |
| PCK2 | CATCCCAACTCTCGATTTTGTG TTCCCAGAAGTCCTTTGTGTTC | 60 | 226 |
| CXCL1 | TCCTCTCACAGCCGCCAGAC ACCAGGAGCAGGAGCAGCAG | 60 | 113 |
| KIF21B | CCGCACAACGAGGAGCAAGAC CCACCAGGAGAGCCGAGGAAG | 60 | 98 |
| VCL | CCTGAAGGAGAAGTACGACAG GATGTAGACCAGGTCTTGTGTG | 60 | 112 |
| FOS | CTTCCCAGAAGAGATGTCTGTG TGGGAACAGGAAGTCATCAAAG | 60 | 193 |
| HMOX1 | CCTCCCTGTACCACATCTATGT GCTCTTCTGGGAAGTAGACAG | 60 | 93 |
| DUSP1 | AGGCCATTGACTTCATAGACTC AGTCCTCATAAGGTAAGCAAGG | 58 | 113 |
| SLC1A1 | GCAGTACAAAACTAAGCGTGAA GTCCCTTTTCTCCCATTTTTCC | 60 | 221 |
| RASA1 | CATCTCCTATTGCTGCAAGAAC GAAGTTCATCTGAATGAGCCAC | 60 | 264 |
| SP1 | TCACTCCATGGATGAAATGACA CAGAGGAGGAAGAGATGATCTG | 58 | 185 |
| ABAT | CATCCGATGACTTCTTTCGGAA CCAGTCATCATCTTCTTGCTGA | 58 | 175 |
| TNFRSF12A | TCTGAGCCTGACCTTCGT GTGGTGAACTTCTCTCTCCTG | 60 | 81 |
| ID3 | CATCCTGTTCTGCCTGCTACTCTTC  ACCAGTCTGTGAGTGGACCAAGG | 60 | 303 |
| BAMBI | GCTGACTTCTTCCAAAGAGTTG GCATTCCAAGTCTAACTTTGCA | 59 | 223 |
| JUNB | CTTCTACCACGACGACTCATAC | 58 | 85 |
|  | TTTCAGGAGTTTGTAGTCGTGT |  |  |
| GAPDH | GCTCGTCGTCGACAACGGCTC CAAACATGATCTGGGTCATCTTCTC | 60 | 167 |
