# Supplementary figures and images for: Genome-Wide Investigation of Genes Regulated by ERα in Breast Cancer Cells
Source: Molecules. 2018 Oct 5;23(10):2543. doi: 10.3390/molecules23102543 (PMC6222792; doi:10.3390/molecules23102543)

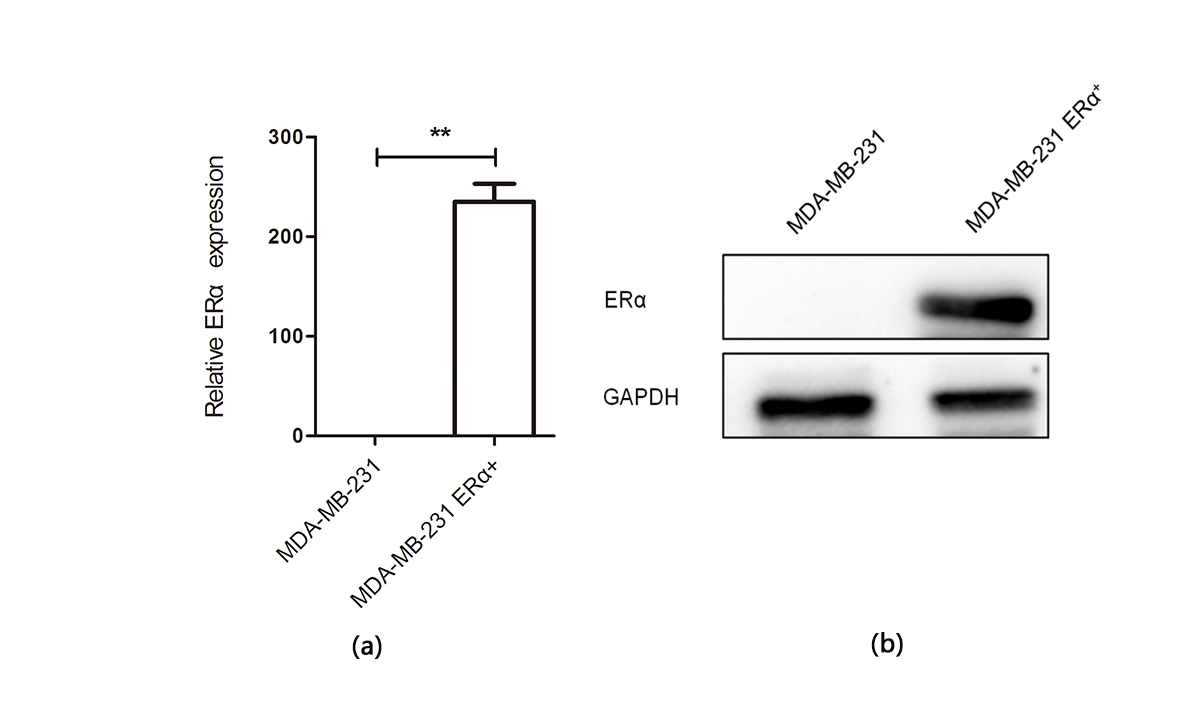

Supplement: Supplementary file 1 [file molecules-23-02543-s001.zip › supplementary/Figure S1.tif]

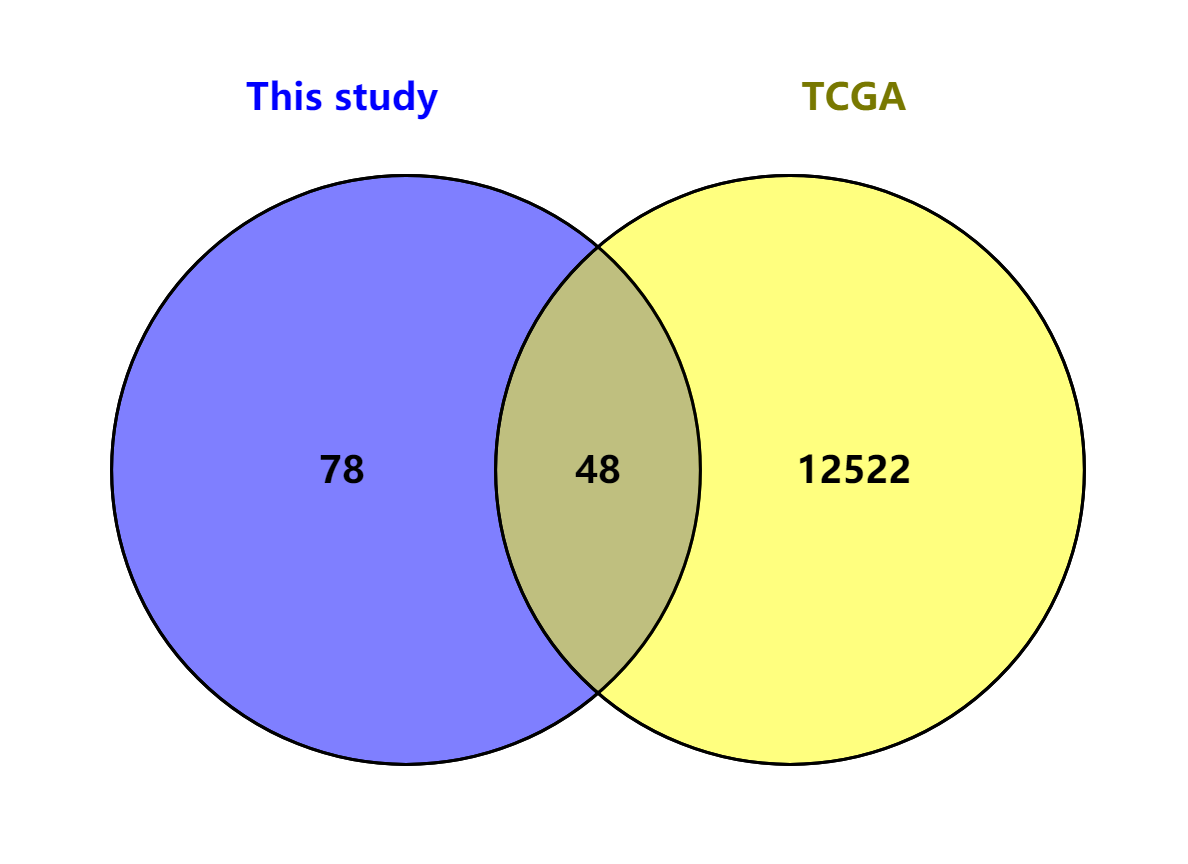

Supplement: Supplementary file 1 [file molecules-23-02543-s001.zip › supplementary/Figure S2.tif]

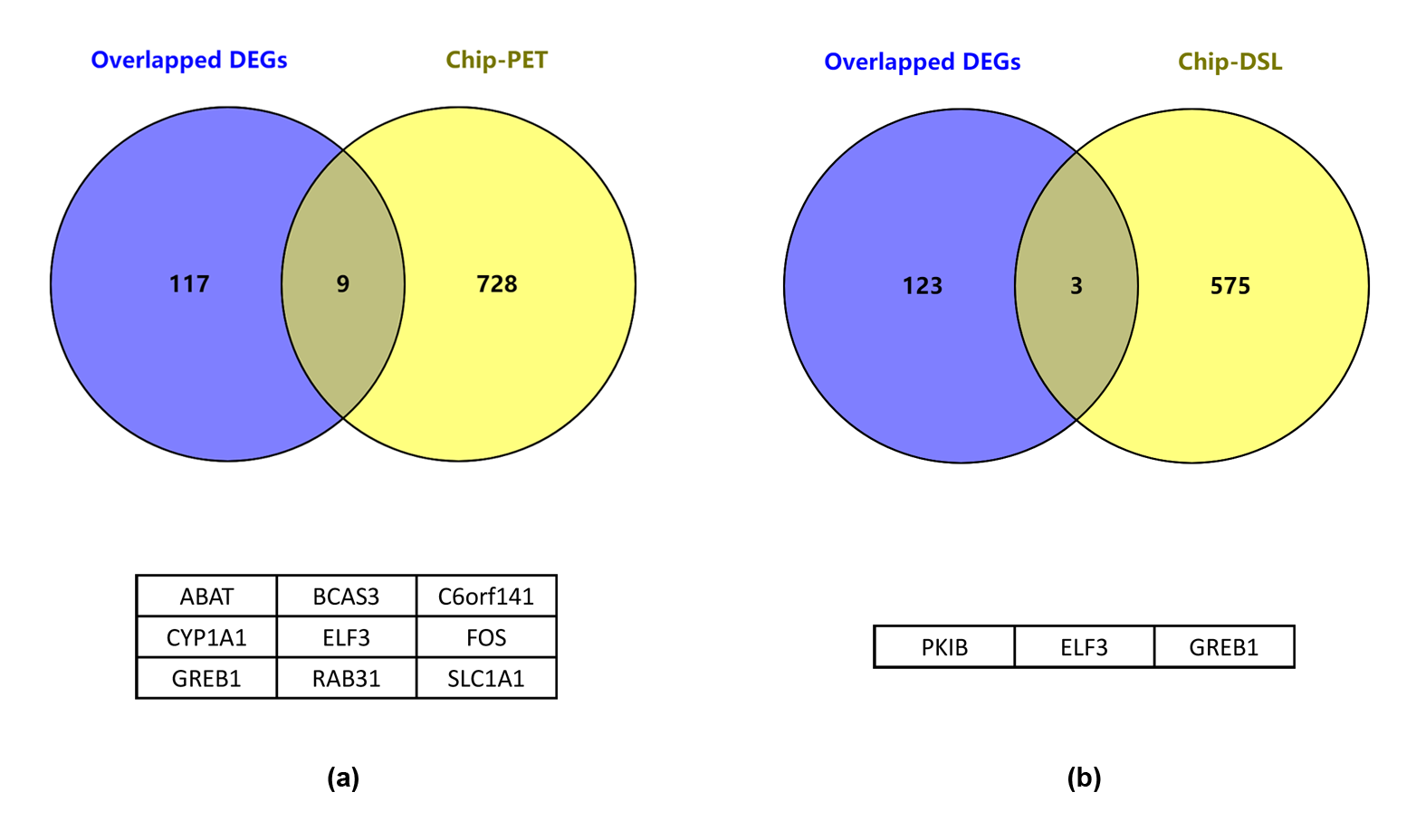

Supplement: Supplementary file 1 [file molecules-23-02543-s001.zip › supplementary/Figure S3.tif]

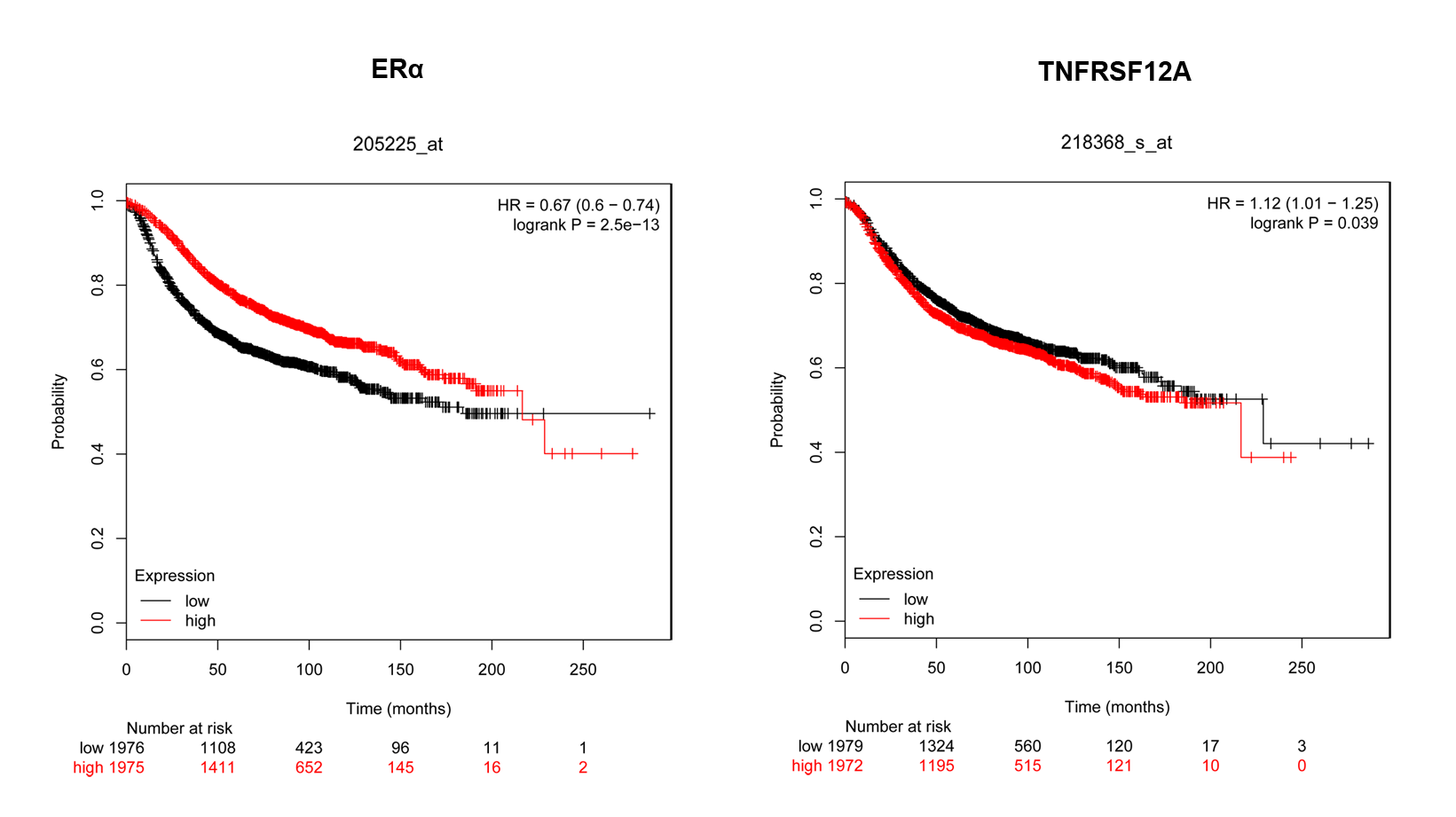

Supplement: Supplementary file 1 [file molecules-23-02543-s001.zip › supplementary/Figure S4.tif]
